# Long-Term Disease Control After locoregional Pelvic Chemoradiation in Patients with Advanced Anal Squamous Cell Carcinoma

**DOI:** 10.3389/fonc.2022.918271

**Published:** 2022-07-22

**Authors:** Athénaïs Grave, Julie Blanc, Berardino De Bari, Mandy Pernot, Fatiha Boulbair, Monique Noirclerc, Angélique Vienot, Stefano Kim, Christophe Borg, Jihane Boustani

**Affiliations:** ^1^ Department of Radiation Oncology, University Hospital of Besançon, Besançon, France; ^2^ Department of Statistics, Centre Georges François Leclerc, Dijon, France; ^3^ Department of Radiation Oncology, Réseau hospitalier neuchâtelois, La Chaux-de-Fonds, Switzerland; ^4^ Department of Radiation Oncology, Nord Franche-Comté Hospital, Montbéliard, France; ^5^ Department of Radiation Oncology, Hasenrain Hospital, Mulhouse, France; ^6^ Department of Medical Oncology, University Hospital of Besançon, Besançon, France

**Keywords:** squamous cell carcinoma of the anus (SCCA), metastatic setting, pelvic chemoradiotherapy, upfront chemotherapy, long-term control

## Abstract

**Introduction:**

The incidence of metastatic squamous cell carcinoma of the anus (SCCA) is increasing. Even if systemic docetaxel, cisplatin, and 5-Fluorouracil (DCF) provide a high rate of long-term remission, the role of pelvic chemoradiation (CRT) is unknown in this setting. We reported the safety and efficacy of local CRT in patients with synchronous metastatic SCCA who achieved objective response after upfront DCF.

**Methods:**

Patients included in Epitopes HPV01 or Epitopes HPV02 or SCARCE trials and treated with DCF followed by pelvic CRT were included. Concurrent chemotherapy was based on mitomycin (MMC) (10 mg/m² for two cycles) and fluoropyrimidine (capecitabine 825 mg/m² twice a day at each RT treatment day or two cycles of intra-venous 5FU 1000 mg/m² from day 1 to day 4). Primary endpoints were safety, local complete response rate, and local progression-free survival (PFS). Secondary endpoints were PFS, overall survival (OS), and metastasis-free survival (MFS).

**Results:**

From 2013 to 2018, 16 patients received DCF followed by a complementary pelvic CRT for advanced SCCA. Median follow-up was 42 months [range, 11-71]. All patients received the complete radiation dose. Compliance to concurrent CT was poor. Overall, 13/15 of the patients (87%) had at least one grade 1-2 acute toxicity and 11/15 of the patients (73%) had at least one grade 3-4 toxicity. There was no treatment-related death. The most frequent grade 3-4 adverse effects were neutropenia (36%), dermatitis (40%), and anitis (47%). Eleven patients (73%) had at least one chronic grade 1 or 2 toxicity. One patient had a grade 4 chronic rectitis (7%). Complete local response rate was 81% at first evaluation and 62.5% at the end of the follow-up. Median local PFS was not reached and the 3-year local PFS was 77% (95%CI 76.8-77).

**Conclusions:**

In patients with metastatic SCCA who had a significant objective response after upfront DCF, local CRT was feasible with high complete local response rate. The good local control rate, despite interruptions due to toxicities and low CT compliance, underline the role of pelvic RT. The high rate of toxicity prompts the need to adapt CRT regimen in the metastatic setting.

## Introduction

Anal squamous cell carcinoma (SCCA) is a rare malignancy with a worldwide incidence of approximately 50,000 new cases and 19,000 deaths in 2020 (1). It represents approximately 3% of all gastro-intestinal cancers, with a predominance in women (2, 3). Its incidence has increased in the last three decades, reflecting its association with Human Papillomavirus (HPV) infection (1, 2). Indeed, 90% of the cases are related to HPV infection, mostly genotype 16 (3). Moreover, the proportions of stage III and stage IV at diagnosis have tripled and doubled, respectively, between 2004 and 2015, as well as the infiltrating lesion rate compared to the *in situ* one (4).

Historically, surgery with abdominoperineal resection was the cornerstone for treatment of localized SCCA, with a 5-year overall survival (OS) rate between 47% and 71%, and a perioperative mortality of 3% (5). Then, the treatment shifted towards organ preservation strategies combining radiation (RT) and chemotherapy (CT) (6), with surgery becoming salvage treatment in case of locoregional failure (7). The ACT-I and EORTC trials have shown the benefit of chemoradiation (CRT) with 5-Fluorouracil (5FU) and mitomycin-C (MMC) compared to RT alone, for local and loco-regional diseases (8, 9). In the update of ACT-I after a 13 year follow-up, CRT was associated with a reduction in the risk of locoregional relapse and an improvement in the relapse-free and colostomy-free survival rates (10). The phase III Radiation Therapy Oncology Group (RTOG) 8704 trial has shown the interest of concurrent MMC in addition to 5FU compared to 5FU alone (11). Later, the EXTRA study demonstrated the safety of capecitabine 825 mg/m² taken orally twice daily with RT instead of intravenous 5FU (12). In these trials, CRT was associated with high rates of acute toxicity, with approximately 30%–50% of grade 3 and 10%–20% of grade 4, especially mucositis, skin toxicity, as well as hematological and gastrointestinal toxicities (9, 11, 13). More recently, the advent of Intensity Modulated Radiation Therapy (IMRT) significantly reduced these toxicities (14).

About 10%–15% of SCCA cases are diagnosed at the metastatic stage (15) and patients are treated with systemic CT. The optimal CT regimen was not clearly defined until 2018 because available data were derived from small retrospective studies (16), including the recommended combination of cisplatin and 5FU (CDDP-5FU) which was based on a small retrospective analysis of 19 patients (17). The addition of docetaxel to cisplatin and 5FU (DCF) showed encouraging results with long-lasting complete responses, including a high rate of pathological complete response (18, 19). Recently, the pooled analysis of Epitopes-HPV01 and 02 studies including 115 patients, has confirmed the modified DCF regimen as the best evidence-based treatment in terms of efficacy and safety (19, 20). The objective response rate obtained with the DCF regimen was 88% and the complete response rate was 40%. Besides, the multicenter randomized non-comparative phase II InterAACT trial evaluated CDDP-5FU with carboplatin plus weekly paclitaxel (CP) in patients with inoperable locally recurrent or metastatic treatment-naïve disease. In this study, CDDP-5FU and CP induced similar objective response rates and progression-free survival. The number of severe adverse events reported was significantly lower in patients exposed to CP (21).

The control of the primary disease is one major issue in advanced SCCA treatment since its progression might significantly hamper the quality of life. However, the role of pelvic CRT in SCCA patients with synchronous metastases in still unknown. In this study, we reported the efficacy and safety of standard CRT in patients with advanced SCCA at diagnosis, who had a significant objective response after DCF as the first line treatment.

## Materials and Methods

### Patients

This retrospective study included patients treated between 2013 and 2018 in three French centers: the University Hospital of Besancon, Nord-Franche Comté Hospital in Montbéliard, and Emile Muller Hospital in Mulhouse. These patients were from the Epitopes-HPV01 (NCT01845779**)** and 02 (NCT02402842) studies (18, 22) as well as SCARCE (NCT03519295) phase II study that evaluated atezolizumab in addition to DCF in metastatic or locally advanced SCCA (23). Patients with a histologically-proven, locally-advanced, or metastatic SCCA, who had an objective response after the upfront CT, were included. The disease was staged according to the 7^th^ TNM classification (24). Objective response was defined as a partial response (PR) or a complete response (CR) on the primary tumor and/or the metastatic sites according to the RECIST criteria version 1.1. if evaluated by Magnetic Resonance Imaging (MRI) and/or computed tomography (CT) scan, and/or according to PERCIST criteria if evaluated by 18F-fluorodeoxyglucose (18F-FDG) Positron Emission Tomography (PET/CT). Patients who received exclusive RT on metastatic sites and those with a contra-indication for RT or concurrent CT were excluded.

### Upfront Chemotherapy

Patients received DCF following two protocols. The standard protocol (sDCF) consisted of docetaxel 75 mg/m² on day 1, cisplatin 75 mg/m² on day 1, and 5FU 750 mg/m² by 24-hour continuous infusion for 5 days, delivered every 21 days. The modified protocol (mDCF) consisted of docetaxel 40 mg/m² on day 1, cisplatin 40 mg/m² on day 1, and 5FU 1200 mg/m² per day for 2 days, every 14 days. In Epitopes-HPV01and Epitopes-HPV02, sDCF was recommended, although not mandatory, for patients aged 75 years or younger and with an ECOG performance status of 0, while mDCF was recommended for those older than 75 years or with an ECOG performance status of 1. In SCARCE, all patients received mDCF. Granulocyte colony-stimulating factor (G-CSF) was administered subcutaneously at the recommended dose of 5 μg/kg per day, for 5 days (for mDCF) to 7 days (for sDCF), as primary prophylaxis of febrile neutropenia for both regimens, as well as to maintain the planned dose intensity.

### Concurrent Chemoradiation

RT consisted of two sequences in most patients. The first sequence delivered 36 Gy in 20 daily fractions of 1.8 Gy to the primary tumor and pelvic lymph nodes. The second sequence delivered 23.4 Gy in 13 daily fractions of 1.8 Gy to the primary tumor, the anal canal, and the residually- involved lymph nodes (total dose of 59.4 Gy in 33 fractions). Treatment was delivered by IMRT, Volumetric Modulated Arc Therapy (VMAT), or tomotherapy. Patients treated until 2016 had a 2-week break between the two sequences. This gap was discontinued starting from the middle of 2016. Residual metastases were treated by surgery or stereotactic RT according to their feasibility. Concurrent CT consisted of two cycles of mitomycin 10 mg/m² at the first and the fifth week of RT, and a fluoropyrimidine-based regimen either with capecitabine 825 mg/m² twice a day each RT treatment day or intra-venous 5FU 1000 mg/m² from day 1 to day 4 of the first and the fifth week of RT.

### Study Objectives

The primary objectives were to evaluate the safety and local efficacy of CRT. The secondary endpoints were metastasis-free survival (MFS), progression-free survival (PFS) and overall survival (OS). Compliance with CT was defined as the delivery of all cycles without dose reduction. Compliance with RT was defined as the absence of interruption related to toxicities. The evaluation of acute (less than 6 months) and chronic (more than 6 months) toxicities was based on CTCAE criteria version 4.03. Objective local response was defined as PR or CR on the primary tumor according to RECIST criteria version 1.1. PFS was defined as the time between the start of DCF and disease recurrence whether local, regional, metastatic, and/or death. Local PFS was defined as the time between the start of DCF and local recurrence, defined by clinical examination and/or imaging (MRI, PET-CT, CT) and/or biopsies. Metastasis-free survival (MFS) was defined as the time between the start of DCF and metastatic recurrence or progression at initial metastatic sites and/or death. OS was defined as the time between the start of DCF and death from any cause.

The first evaluation was performed three months after the end of CRT by MRI and clinical examination. Then, patients were followed every three months by CT scan and clinical examination for the first two years, every six months by CT scan, and clinical examination until the fifth year and once per year thereafter.

### Statistical Methods

The distribution of each continuous variable was summarized by its median and range. The distribution of each categorical variable was summarized in terms of its frequency and percentage. For patients alive and/or progression-free, the actual date of the last follow-up visit was used as the end date. Survival curves were generated using the Kaplan–Meier method.

## Results

### Patients’ Characteristics

Overall, 16 patients treated between 2013 and 2018 were included. Patients’ characteristics are shown in **Table 1** and in **Supplementary Table 1**. The two patients from the SCARCE trial received mDCF without atezolizumab. The median age was 58.5 years [range, 45-76]. Patients were predominantly women (81%). There was 69% of stage T4, 75% of stage N3, and 75% of stage M1 disease. SCCA was moderately differentiated in half of the cases. One patient presented with a neuro-endocrine component. All patients had either p16+ or HPV+ disease, except for one patient with missing data. One patient was HPV positive and p16 negative with HPV 6 genotype. All patients had negative Human Immunodeficient Virus (HIV) blood tests.

**Table 1:** Patients’ characteristics.

		Number (%)
**Gender**		
	Male	3 (19)
	Female	13 (81)
**Age (years)**		
	Median [range]	58.5 [45;76]
**Center**		
	Besancon	8 (50)
	Montbeliard	6 (37.5)
	Mulhouse	2 (12.5)
**Initial study**		
	Epitopes-HPV01	9 (56)
	Epitopes-HPV02	5 (31)
	SCARCE	2 (13)
**Performance status**		
	0	9 (56)
	1	6 (38)
	2	1 (6)
**T stage**		
	1	0
	2	2 (12)
	3	3 (19)
	4	11 (69)
**N stage**		
	0	0
	1	2 (12.5)
	2	2 (12.5)
	3	12 (75)
**M stage**		
	M0	4 (25)
	M1	12 (75)
**Stage at initial diagnosic**		
	IIIB	4 (25)
	IV	12 (75)
**Metastatic site**		
	Liver	7 (44)
	Lung	2 (12.5)
	Bone	2 (12.5)
	Commun iliac nodes	1 (6)
	Skin	1 (6)
**Histology**		
	Well differentiated	2 (12.5)
	Moderately differentiated	8 (50)
	Poorly differentiated	2 (12.5)
	Undifferentiated	1 (6)
	Other	1 (6)
	Unknown	2 (12.5)
**HPV and p16 status**		
	Positive	15 (94)
	Negative	0
	Unknown	1 (6)

### Upfront Chemotherapy

The characteristics and response to upfront CT are shown in **Table 2A**. mDCF was prescribed in 75% of the patients. The median numbers of administrated cycles were 6 [range, 3-10] for sDCF and 8 [range, 5-11] for mDCF. After CT, 75% of the patients had a PR and 25% had a CR on the primary tumor (**Table 2A**). One patient had a dissociated response with a lymph node progression, a PR on the primary tumor, and a CR on a single liver metastasis. Available toxicity data are reported in **Table 2B**. Toxicity data were available for 14 patients. Overall, 12 patients (86%) had at least one grade 1-2 toxicity and 5 patients (36%) had at least one grade 3-4 toxicity. The most frequent side effects were anemia, neutropenia, peripheral neuropathy, nausea/vomiting, diarrhea, and asthenia. There was only one patient with a hematological grade 4 toxicity (anemia and neutropenia). There was no grade 4 non-hematological toxicity and no grade 5. After DCF, eight patients (50%) underwent a tumor ablation of their residual metastases before CRT. Indeed, six patients (37.5%) had liver metastasectomy before CRT. Among them, one patient (6.25%) had also stereotactic RT on the third lumbar vertebra. For two patients (12.5%), the metastatic site was included in the RT field (common iliac lymph node (n=1) and right ischio-pubic branch (n=1)).

**Table 2a:** Response and toxicities after upfront chemotherapy.

Response after upfront chemotherapy.	
**DCF protocol, n(%)**	
Standard	4 (25)
Modified	12 (75)
**Number of cycles, median [range]**	
sDCF	6 [3;10]
mDCF	8 [5;11]
**Primary tumor response, n(%)**	
CR	4 (25)
PR	12 (75)
**Metastasis response, n(%)**	
CR	7 (59)
PR	4 (33)
PD	1 (8)
**Global response, n(%)**	
CR	3 (19)
PR	12 (75)
SD	0
PD	1 (6)

DCF, Docetaxel, Cisplatin and 5-Fluorouracil; sDCF, standard DCF; mDCF, modified DCF; CR, complete response; PR, partial response; SD, stable disease; PD, progressive disease.

**Table 2b:** Response and toxicities after upfront chemotherapy.

Chemotherapy-induced toxicities.		
	**Grade 1-2, n (%)**	**Grade 3-4, n (%)**
Hematological toxicities*		
Anemia	4 (27)	1 (8)
Neutropenia	2 (15)	3 (23)
Thrombopenia	2 (15)	0
Febrile neutropenia	0	2 (15)
Non-hematological toxicities**		
Nausea/vomiting	5 (36)	2 (50)
Asthenia	7 (50)	1 (7)
Anorexia	3 (21)	0
Mucositis	3 (21)	1 (7)
Neuropathy	8 (57)	0
Diarrhea	4 (29)	1 (7)
Dysgeusia	2 (14)	0
Hand-foot syndrom	3 (21)	0

*Calculated for 13 patients (missing data for three patients).

**Calculated for 14 patients (missing data for two patients).

Data n (%) are indicated for which a patient could have more than one adverse event.

### Compliance with CRT

Treatment characteristics for patients with assessable RT plans are displayed in **Table 3**. The median time between the end of CT and the start of CRT was 60.5 days [range, 17-97]. IMRT was used in 75% of the patients, Volumetric Modulated Arc Therapy (VMAT) in two patients (12.5%), and Tomotherapy in two patients (12.5%) All patients received the scheduled RT dose of 59.4 Gy with the exception of one patient who received 60 Gy in 30 fractions. Thirteen patients (81%) received a prophylactic 36 Gy to the pelvic lymph nodes. The other three patients received higher doses with respectively 40 Gy, 46 Gy and 50 Gy. For patients with available data (n=12), irradiated lymph nodes included the external and internal iliac, presacral, inguinal, and perirectal areas. Three patients (19%) were also irradiated on common iliac lymph nodes. The median overall treatment time (OTT) was 50.5 days [range, 42-63]. A treatment gap after the first sequence was scheduled in four patients and the median OTT in these patients was 59 days [range, 57-60]. RT had to be interrupted because of acute toxicity in four patients (25%), one of which also had a scheduled gap. Nine patients (56%) completed their RT without interruption. The median OTT in these patients was 47 days [range, 42-52].

**Table 3:** Chemoradiation characteristics.

Time between end of chemotherapy and start of CRT (days), median [range]	
All	60.5 [17;97]
Patients treated on metastasis before CRT	73.5 [59;76]
Patients not treated on metastasis before CRT	40 [17;97]
**Total dose (Gy) on anal canal and positive nodes, n (%)**	
60	1 (6)
59.4	15 (94)
**Radiation technique, n (%)**	
IMRT	12 (75)
VMAT	2 (12.5)
Tomotherapy	2 (12.5)
**Dose to the pelvic nodes (Gy), n (%)**	
36	13 (81.25)
40	1 (6.25)
46	1 (6.25)
50.01	1 (6.25)
**Irradiated nodes, n (%)**	
Common iliac	3 (19)
External iliac	12 (75)
Internal iliac	12 (75)
Presacral	12 (75)
Inguinal	12 (75)
Perirectal	12 (75)
Missing data	4 (25)
**Dose by fraction (Gy), n (%)**	
2	1 (6)
1.8	15 (94)
**Number of fractions, n (%)**	
30	1 (6)
33	15 (94)
**Overall treatment time (days), median [range]**	
All	50.5 [42; 63]
With gap	59 [57; 60]
With interruption for toxicities	57 [52; 63]
Without any interruption	47 [42; 52]
**Number of cycles of Mitomycin, n (%)**	
1	8 (50)
2	7 (44)
Missing data	1 (6)
**Capecitabine or intravenous 5FU adaptation, n (%)**	
Yes	8 (50)
No	4 (25)
Missing data	4 (25)
**Reason for radiation interruption, n (%)**	n (%)
No interruption	9 (56)
Gap	4 (25)
Toxicity	4 (25)

CRT, chemoradiation; IMRT, Intensity Modulated Radiation Therapy; VMAT, Volumetric Modulated Arc Therapy; 5FU, 5-Fluorouracil.

Compliance with concurrent CT is detailed in **Supplementary Table 2**. Fourteen patients (88%) received the first cycle of MMC. Only 38% (6/16) of the patients received the complete dose of second cycle of MMC and 31% (5/16) received the complete dose of 5FU. The main reasons for concurrent CT adaptation or interruption were hematological toxicity and one case of hand-foot syndrome.

### Toxicities Related to CRT

Toxicity data were available in 15 patients (**Table 4**). Regarding acute toxicities, 13 patients (87%) had at least one grade 1-2 toxicity and 11 (73%) had at least one grade 3-4 toxicity. There was no grade 5 toxicity. The most frequent side effects were neutropenia (36%), dermatitis (40%), and anitis (47%) (**Table 4A**). Among acute non-hematological toxicities (n=49), 43% were grade 1, 28.5% grade 2, 28.5% grade 3 and no grade 4. Among acute hematological toxicities (n=21), there were 14% grade 1, 33% grade 2, 38% grade 3, and 14% grade 4.

**Table 4A:** Toxicities related to chemoradiation.

Acute toxicities				
	G1	G2	G3	G4
Non-hematological toxicities*, n(%)				0
Skin	3 (20)	3 (20)	6 (40)	0
Diarrhea	3 (20)	5 (33)	0	0
Colitis	2 (13)	0	0	0
Urinary advese events	5 (33)	0	1 (7)	0
Anitis	2 (13)	1 (6.25)	7 (47)	0
Asthenia	2 (13)	2 (13)	0	0
Pelvic pain	3 (20)	2 (13)	0	0
Nausea	1 (7)	1 (7)	0	0
Hematological toxicities**, n(%)				
Anemia	1 (7)	2 (14)	1 (7)	1 (7)
Neutropenia	0	2 (14)	5 (36)	0
Thrombopenia	2 (14)	3 (31)	1 (7)	2 (14)
Lymphopenia	0	0	1 (7)	0

*analysis on 15 patients (missing data for one patient).

**analysis on 14 patients (missiong data for two patients).

Regarding chronic toxicities, 11 patients (73%) had at least one grade 1 or 2 toxicity (**Table 4B**). One patient (7%) with history of cirrhosis had a grade 4 chronic rectitis, which led to several hospitalizations, multiple transfusions, two endoscopic hemostasis gestures, and hemorrhagic shock. One patient had an asymptomatic rectovaginal fistula that did not require any intervention. Among chronic toxicities (n=21), there were 71.4% grade 1, 19% grade 2, and 9.5% grade 4.

**Table 4b:** Toxicities related to chemoradiation.

Chronic toxicities***				
N(%)	G1	G2	G3	G4
Incontinence	3 (20)	1 (7)	0	0
Imperiosity	1 (7)	1 (7)	0	0
Telangectasia	1 (7)	0	0	0
Fibrosis	4 (27)	0	0	0
Anal stricture	0	1 (7)	0	0
Asthenia	2 (13)	0	0	0
Diarrhea	1 (7)	0	0	0
Rectitis	3 (20)	0	0	1 (7)
Anemia	0	0	0	1 (7)
Lymphopenia	0	1 (7)	0	0

***analysis on 15 patients because one patient died four months after the end of CRT.

### Response to CRT and Survival Outcomes

**Figure 1:**
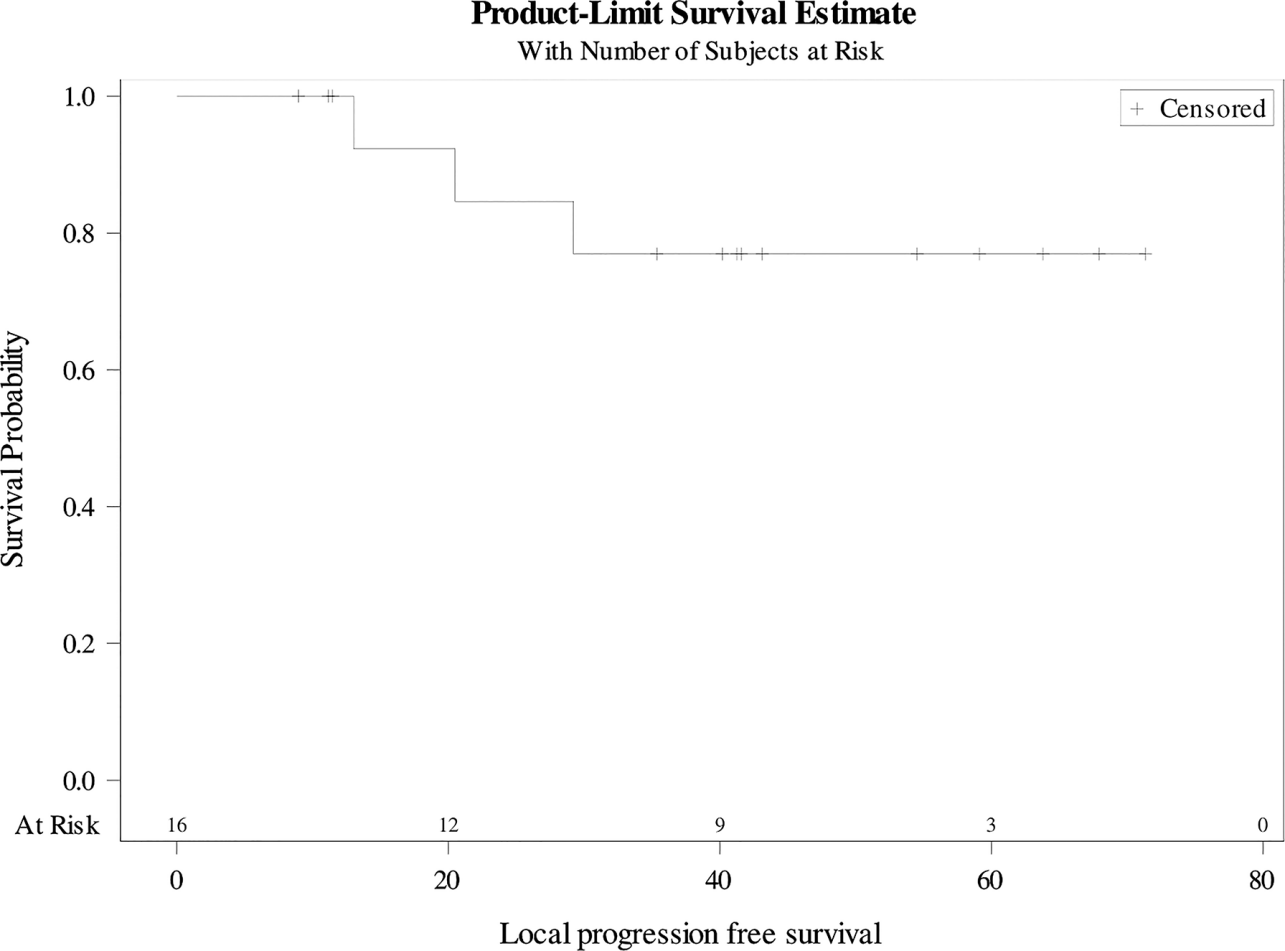
Local progression-free survival.

The median follow-up was 42 months [11-71]. All patients presented an objective local response at the first evaluation (**Table 5**). Among them, 13 (81%) were in local CR and three in local PR (19%). Only one patient (6%) had a global PD at the first evaluation with a metastatic progression. At the last follow-up, 10 patients (62.5%) were still in local CR. The 3-year local PFS rate was 77% (95%CI 76.8-77) (**Figure 1**). Median local PFS was not reached. Three patients in global CR after DCF were still in global CR after CRT. One patient in local CR and metastatic PR after DCF, was in global CR after CRT (**Supplementary Table 1**).

**Table 5:** Response after chemoradiation.

Local response	N (%)
CR	13 (81)
PR	3 (19)
SD	0
PD	0
**Metastatic response**	**N (%)**
CR	13 (81)
PR	0
SD	0
PD	3 (19)
**Global response**	**N (%)**
CR	11 (69)
PR	2 (12)
SD	0
PD	3 (19)

CR, Complete response; PR, Partial response;

SD, Stable disease; PD, Progressive disease.

Among the twelve patients in local PR after DCF, nine (75%) were in CR, and three (25%) were in PR after CRT (**Supplementary Table 1**). Among patients with local PR (n=3) after CRT, two received a second line CT and one had a complete resection of a locally residual tumor. Three patients (19%) presented a local recurrence at 7, 13, and 22 months after the end of CRT, respectively. Among them, two patients (12%) received a salvage abdominal resection (one was in complete resection with only 10% of tumor residue (ypT1m), the other with an infra-millimetric margin (ypT2 (bifocal)), and one of them received a second line CT. Nine patients (56%) presented a systemic progression (three hepatic, two pulmonary, one peritoneal, two on lymph nodes, and one generalized). Among them, one presented a single pulmonary metastasis 15 months after CRT that was treated by stereotactic RT. This patient was in CR 56 months after the start of DCF. Response and treatments at progression are detailed in **Supplementary Table 1**

**Figure 2:**
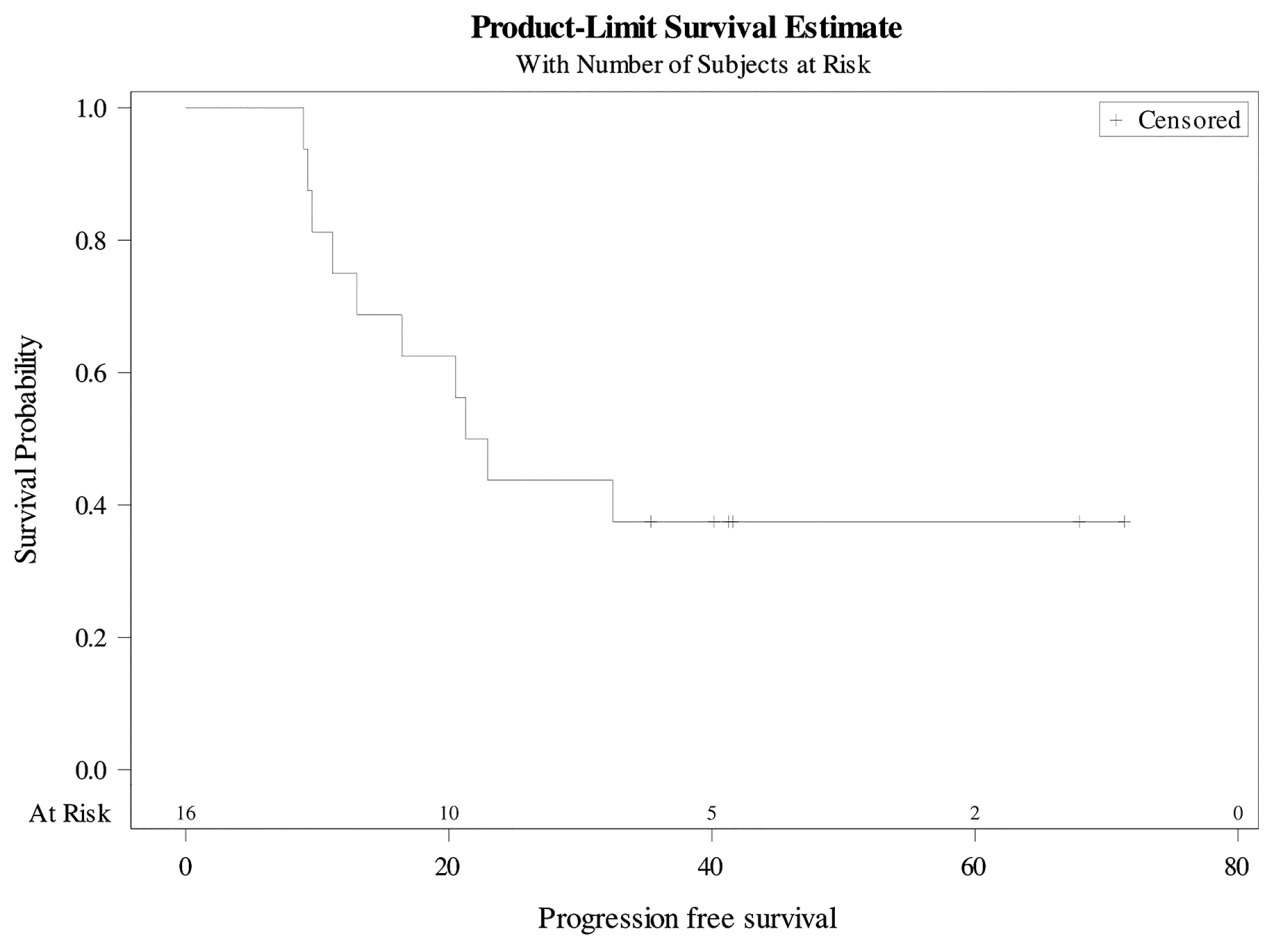
Progression-free survival.

**Figure 3:**
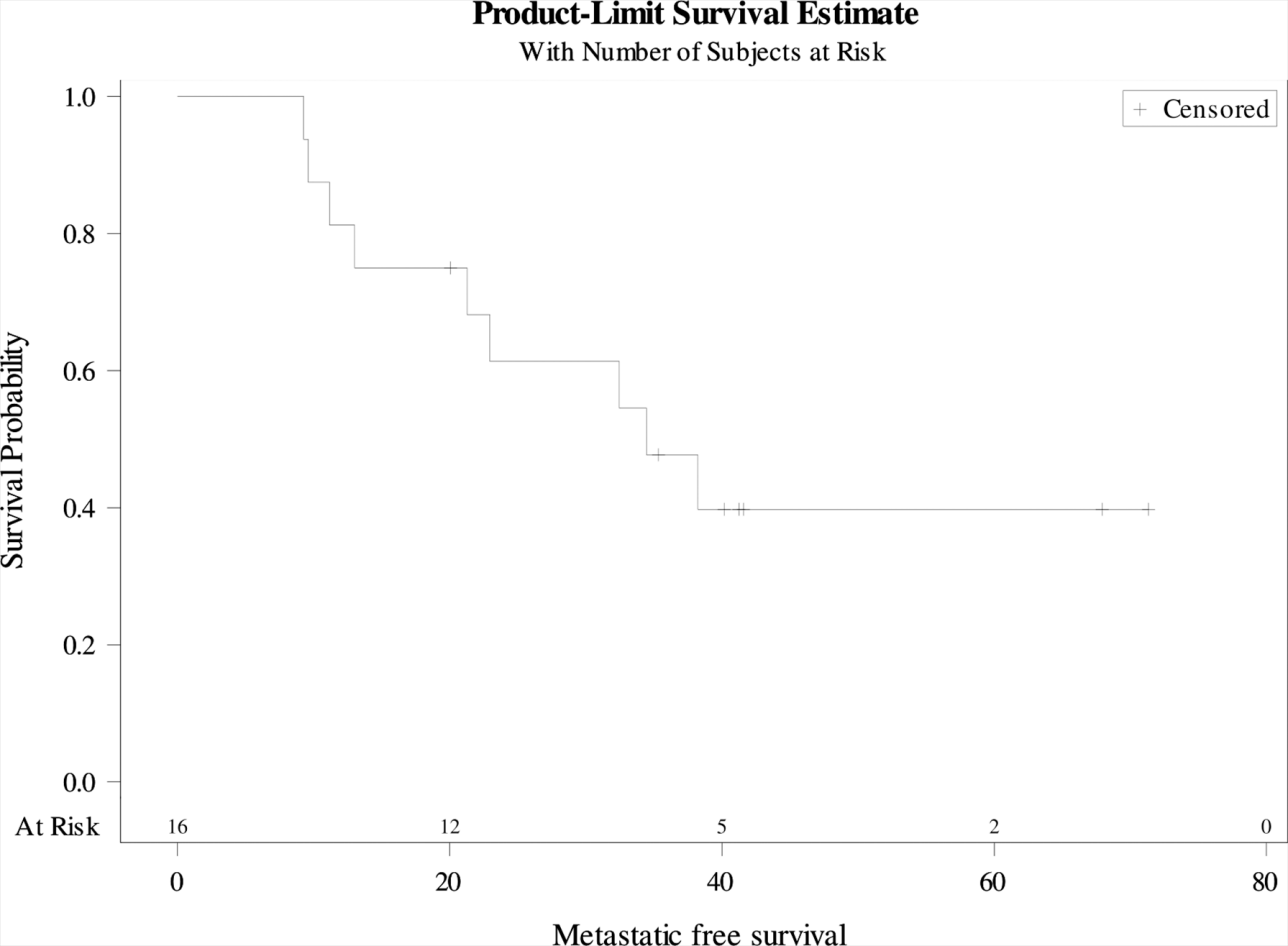
Metastatic-free survival.

**Figure 4:**
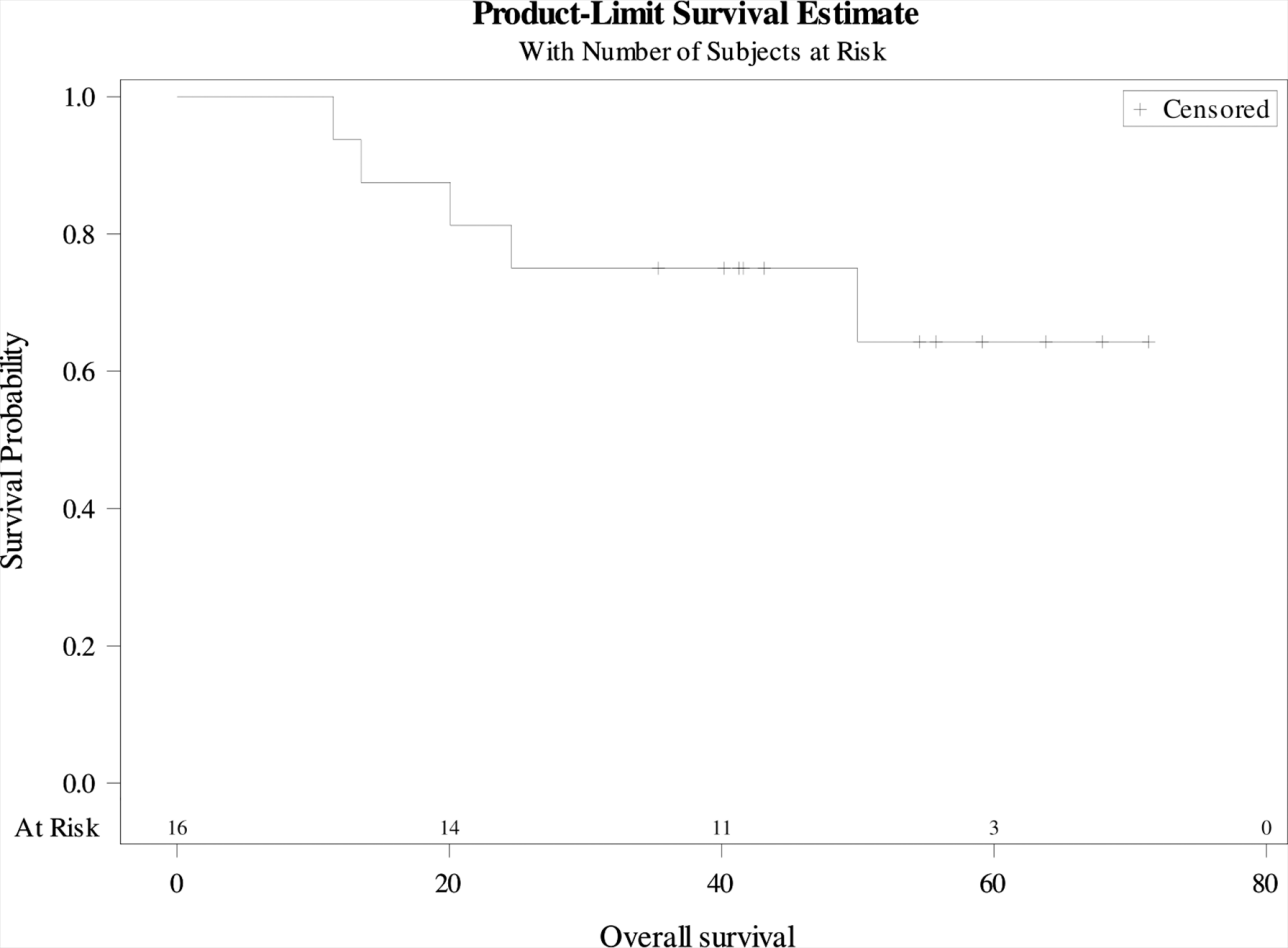
Overall Survival.

Overall, the median PFS was 22 months [9-71] (**Figure 2**). The 3-year PFS rate was 37.5% (95%CI 25.4-49.6). The median MFS was 34 months [9-71] (**Figure 3**). The 3-year MFS rate was 48% (95%CI 34.8-60.6). Overall, five patients died. The median OS was not reached (**Figure 4**). The 3-year OS rate was 75% (95%CI 64.2-85.8).

## Discussion

In this retrospective analysis, we showed that CRT after DCF was feasible in advanced SCCA with complete local responses in 81% of patients at first evaluation. Treatment-related toxicities were frequent and compliance with concurrent CT was low.

A major concern about the DCF regimen is its toxic effects at the standard dose. Previous studies have shown better compliance and safety profile in patients treated with mDCF (19, 22, 25). In this study, most of the patients received eight cycles of mDCF. Overall, 86% of patients presented at least one grade 1-2 toxicity and the grade 3-4 toxicity rate was 36%. This was lower than in Epitopes-HPV02 where the rate of grade 3-4 toxicity was 83% with sDCF and 53% with mDCF (22). We found a two-month delay between the end of DCF and the start of CRT. This can be explained in part by CT-related toxicities. Indeed, the longest delay of 97 days was due to a hematological grade 4 toxicity. Also, this can be partially explained by the fact that some metastatic sites were treated in the interval, thus delaying the start of CRT. It is noteworthy that increasing total treatment time did not contribute to treatment failure since most of the patients had a global objective response. Carboplatin plus paclitaxel (CP) is another validated regimen in metastatic SCCA. However, the grade 3-4 toxicity rate is higher (71%) including hematological toxicities (29% of grade 3-4 neutropenia, 10% of grade 3-4 anemia, and 5% of febrile neutropenia) (21). Hence, even though there is no head-to-head comparison between these two prospectively validated regimens, mDCF has shown a higher response rate with lower toxicity (26, 27).

Compliance to RT was high since all patients received the planned dose and toxicity-related interruptions occurred in only four patients. However, the compliance to concurrent CT was poor, with 38% (6/16) of the patients receiving two cycles of MMC at full dose, and 31% (5/16) receiving a complete dose of 5FU. Conversely, previous studies reported compliance rates that ranged between 68% and 75% in patients treated with 5FU+MMC-based CRT (12, 28) and approximately 90% in patients treated with a 2-week gap (13, 29). The low compliance rate can be explained by the high rate of hematological toxicities that might have been fostered by the upfront CT. Hematological acute toxicities were frequent with 36% of grade 3 neutropenia and 14% of grade 4 thrombocytopenia. The hematological toxicity related to concurrent CT and RT depends on several factors such as the total RT dose, the volume of irradiated bone marrow, the type and dose of CT, and the medullary reserve of hematopoietic capacity (30). Patients who have received prior CT often exhibit irreversible chronic bone marrow damage producing impaired hematopoietic reserve and function (31). In Flam et al. the addition of MMC to 5-FU based RT translated into a benefit in colostomy-free survival but with no effect on OS (11). These results suggest the possibility of delivering one cycle of MMC instead of two, or even omitting MMC, without compromising survival. Conversely, the hematological adverse effects might also result from the importance of the volume of irradiated bone marrow. Improvements in RT delivery techniques, such as IMRT, have led to fewer gastrointestinal and hematological toxicities compared with 3-dimensional conformal radiation therapy (3DCRT) (14). Despite these improvements with IMRT, hematological toxicities remain a significant cause of treatment interruption. These findings underline the importance of minimizing the volume of bone marrow exposed to ionizing radiations and the need to integrate dosimetric constraints to the pelvic bones into IMRT planning. Another potential option that could improve the compliance and safety of CRT might be to reduce the treated volumes by limiting the irradiation to initially involved lymph nodes and residual disease, instead of irradiating all the lymphatic regions. Moreover, we can hypothesize that lowering the total dose delivered to the primary tumor site may be enough in patients who are in complete response or significant partial response on the primary tumor after DCF. In patients with localized SCCA for instance, the ACT 4 trial is currently comparing standard-dose CRT (50.4Gy in 28 fractions) with reduced-dose CRT (41.4Gy in 23 fractions) in patients with intermediate-risk disease (32). A national cancer database review included 582 patients who had metastatic SCCA treated with pelvic CRT (any dose) or CT alone. Treatment details were not specified and there was no data regarding PFS, local control, and toxicity. Nonetheless, pelvic CRT was associated with improved survival in comparison with CT alone, despite the fact that RT could have been at curative or palliative dose (33).

The nature of the non-hematological acute toxicities in our series was in line with those observed in other studies (8, 9, 11, 12, 28). Grade 3-4 skin acute toxicities were frequent and as expected (12). Rates of diarrhea were also similar (around 55%) but rates of nausea were lower in our study (12.5% versus 58%). This could be explained by IMRT planning versus 3D in EXTRA (12). In the EORTC the trial, there was only 30% of grade 3 and no grade 4 but all the patients had a gap (29).

Conversely, the role of induction CT has been previously studied in the non-metastatic setting. Two phase 3 trials evaluated the role of induction CDDP-5FU-based CT before CRT in anal cancer (34, 35). In ACCORD 03, patients received either CRT alone (45Gy in 25 fractions + 15-25Gy boost after a 3-week gap and concurrent CDDP-5FU) or with two cycles of CDDP-5FU-based induction CT (34). Compliance rates were 98% for RT and 93% for concurrent CT. Acute grade 3-4 toxicity was observed in 73 patients (24%; 14% hematological, 9% non-hematological) receiving induction CT and 46 patients (15%; 6% hematological, 9% non-hematological) without induction CT. In RTOG 98-11, patients received either RT (45-59 Gy) + 5FU-MMC or two cycles of induction 5FU-CDDP followed by RT (45-59 Gy) + 5FU-CDDP (35). RT adherence was 91% in the MMC-based group and 88% in the CDDP-based group. CT adherence was 95% in the MMC-based group and 94% in the CDDP-based group. Grade 3-4 acute toxicity rates were similar for non-hematological events (74%). Hematological side effects were more frequent in MMC-based group (61% versus 42%; p<0.001). In the RTOG 98-11 trial, there were 74% grade 3-4 non-hematological acute toxicities and 42% grade 3-4 hematological acute toxicities as compared to 93% and 79%, respectively, in our series. The difference in hematological toxicity rates compared to our data could be explained by the number of CT cycles before CRT, as well as the addition of Docetaxel that can induce high rates of neutropenia.

In this study, the rate of complete local response at first evaluation after CRT was 81%. The rates of complete response ranged between 65%–90% in most of the studies (8, 9, 12, 28, 36). Our rate was lower than the highest reported one in ACTII and EXTRA, but these studies included very few advanced tumors (13% T4 and 30% N+ in ACTII, 4% T4 and 8% N+ in EXTRA). In fact, in locally advanced stages with T3/T4 or with N+ disease, the complete response rate was about 65%–70% (12, 28). Moreover, in our study, the first evaluation was performed at 3 months, which can be premature since responses can be seen up to 26 weeks after CRT (37). These findings showed good local control despite the gap, the interruptions due to toxicities, and the low compliance with CT, underlining the role of pelvic RT in the metastatic setting.

Interestingly, patients with local progression were compliant with concurrent CT, but they all had an interruption of RT. Among them, two were treated before 2016 with a gap and the third presented a grade 3 acute skin toxicity that led to treatment interruption. It is now well-established that increasing the OTT has a negative impact on survival and that gaps should be avoided (38, 39). Therefore, in patients treated with a gap, the toxicity rates and efficacy of CRT could have been underestimated.

Overall, 37.5% of the patients were in complete remission, with the longest follow-up of 71 months. In the Epitopes-HPV pooled analysis, the complete remission rate was 40.3%, and the median PFS was 12.2 months (versus 14.9 months in our study) with a 3-year PFS rate of 26% (versus 33% in our study) (19). However, this is not a head-to-head comparison and should be interpreted with caution. Results of principal studies for metastatic SCCA treated with CT alone or with pelvic RT are summarized in **Supplementary Table 3** (16, 17, 19, 21, 25, 33, 40–43).

We reported more metastatic progression (56%) than local progression (33%). This underlines the need for enhancing not only the local control but also the distant control in SCCA. Treatment combinations with immunotherapy are being tested. Anti-PD-1/PD-L1 antibodies are potential candidates since long‐lasting complete responses have been seen in chemorefractory patients in advanced SCCA (44). The association of DCF and an anti‐PD1/L1 is feasible, with no particular safety signal in the SCARCE trial in the metastatic setting, which evaluated DCF with or without atezolizumab (23). The phase II INTERACT-ION is now evaluating the efficacy and safety of DCF with ezabenlimab, an anti‐PD1 antibody, as neoadjuvant treatment in stage III SCCA patients treated with CRT (NCT04719988).

Over the past decade, several studies have provided evidence that in oligometastatic disease, treating the metastatic sites confers a survival advantage. Recently, the phase II SABR-COMET trial recruited patients with any primary histology, metachronous oligometastatic disease, and previously-treated primary disease (45). The trial has shown an increased OS in patients who received stereotactic treatment to sites of metastases compared with palliative care alone, supporting the survival benefit of consolidation treatment in the context of oligometastatic disease. In SCCA with oligometastatic disease, the SPARTANA trial will evaluate the interest of the addition of stereotactic RT of metastatic sites to mDCF and spartalizumab, an anti-PD1 antibody. Local CRT will be performed in the case of synchronous disease (NCT04894370) (46).

Conversely, data have shown that the primary tumor may release factors that create a tumor microenvironment that favors the capacity of tumor cells to metastasize. Moreover, circulating tumor cells can seed not only to regional and distant sites but also to the primary tumor itself. This prompted clinicians to treat the primary tumor even in the metastatic setting. The addition of local treatment to the primary tumor translated into improved PFS and/or OS in several cancer types such as prostate, breast, or lung cancer, especially in the oligometastatic setting (26, 47–52). In a series from MD Anderson Hospital, 33 patients with oligometastatic SCCA amenable to locoregional therapy (surgical resection or CRT to limited sites) were analyzed. A significant survival benefit (53 versus 17 months; p <0.001) was seen in comparison with CT alone approaches (16). However, local strategies included treatment of metastases by surgical resection or radiofrequency ablation in 60% of the patients and pelvic CRT in only 40% of the patients. In one small series, 6 patients with anal canal cancer with synchronous para-aortic nodal metastases were treated with definitive CRT. Three-year local control and OS rates were 100% and 63%, respectively (16). Contrary to our study, there was no upfront CT and no visceral metastasis. In another series, 50 patients with metastatic SCCA treated by combining systemic CT and local ablative treatment to remove all metastases through surgery, radiofrequency ablation, or RT, had a median overall survival of 22.0 months (95% CI, 15.3–28.6) versus 13.0 months (95% CI, 9.5–16.5; p = 0.002) in those without ablative treatment (40). Recently, a series of 1,457 patients treated with CRT for metastatic anal cancer showed a benefit in survival compared to CT alone (43). However, in this National Cancer Database, there was a heterogeneity in treatments and information on concurrent CT regimen, and irradiation scheme and toxicity information was not available.

As shown in **Supplementary Table 1**, only four patients were in local complete response after upfront CT. These patients were included in the Epitopes HPV01 or Epitopes HPV02 trials in which complementary local treatments were allowed after the planned DCF cycles at the discretion of each local institutional multidisciplinary board. Despite local complete control being achieved after DCF, these patients received CRT probably to reduce the risk of pelvic recurrence. In fact, most patients would experience symptoms of pain, bleeding, or obstruction, which can significantly alter their quality of life and lead to life-threatening complications. In Wang et al., therapeutic RT defined by a dose >45Gy (with doses ranging between 45 and 67.8Gy) was associated with better survival than palliative RT (43) so there is probably an impact of the RT dose. Furthermore, the seed-and-soil theory provides a biologic rationale for definitive RT of the primary site even after a local complete response. Hence, eradication of the primary tumor and the micrometastatic environment through definitive RT would decrease the spread of tumor cells to distant sites and thus, the appearance of new metastases.

The principal limitations of our study are the small size and its retrospective nature with missing data. Also, there was no comparison with CT alone. However, few patients are eligible for such an aggressive strategy. Data from retrospective series remain instructive and help to guide the management of this group of patients, in the absence of level I evidence. The selection of appropriate patients will probably be the greatest challenge in defining the role of primary site treatment. Doing so may involve a number of factors, including clinical and radiological parameters, as well as biomarkers. The level of circulating tumor DNA (ctDNA) has been shown in many cancers to be associated with tumor response (53) and the detection of HPV ctDNA at metastatic stage after DCF was associated with a poor PFS in SCCA (54). The prognosis value of Monocytic‐MDSC in SCCA patients treated by DCF was observed in two clinical trials (55). After DCF, the percentage of M‐MDSC diminished and high M‐MDSC levels were significantly associated with shorter PFS and OS at baseline and after CT. These findings suggest that local CRT might not be an optimal option in patients with detectable HPV ctDNA or high M‐MDSC levels after upfront CT. However, their utility in patient selection to improve outcomes still needs to be evaluated in clinical trials.

## Conclusion

In patients with metastatic SCCA who had a significant objective response after upfront DCF, local CRT was feasible with a high complete local response rate and significant long-term disease control. Acute toxicities were frequent with 73% of the patients presenting with at least one grade 3-4 adverse event. The compliance with concurrent CT was low. The good local control rate, despite interruptions due to toxicities and low CT compliance, underline the role of pelvic RT. The modalities of CRT in terms of volume, dose, and concurrent CT may need adaptation for tumors in complete or partial response in the metastatic setting in order to maintain a high local control without compromising quality of life. These findings warrant confirmation in prospective studies, including studies of the optimal patient population for pelvic CRT and the optimal treatment regimen.

## Data Availability Statement

The raw data supporting the conclusions of this article will be made available by the authors, without undue reservation.

## Ethics Statement

The studies involving human participants were reviewed and approved by French Committee for Protection of Persons. The patients/participants provided their written informed consent to participate in this study.

## Author Contributions

SK and CB conceived of, designed the study, and analyzed the data. AG and JBo analyzed the data, wrote the manuscript and edited the manuscript. BB, MP, FB, AV, and MN performed the treatments and analyzed the data. JBl was responsible for the statistics. All authors contributed to the article and approved the submitted version.

## Conflict of Interest

The authors declare that the research was conducted in the absence of any commercial or financial relationships that could be construed as a potential conflict of interest.

## Publisher’s Note

All claims expressed in this article are solely those of the authors and do not necessarily represent those of their affiliated organizations, or those of the publisher, the editors and the reviewers. Any product that may be evaluated in this article, or claim that may be made by its manufacturer, is not guaranteed or endorsed by the publisher.
