# Supplementary material for: Long-Term Disease Control After locoregional Pelvic Chemoradiation in Patients with Advanced Anal Squamous Cell Carcinoma
Source: Front Oncol. 2022 Jul 22;12:918271. doi: 10.3389/fonc.2022.918271 (PMC9354951; doi:10.3389/fonc.2022.918271)
Supplement: Supplementary file 1 [file Table_1.docx]

**Supplementary Table 1.** Detailed patients’ characteristics

|  |  |  |  |  |  |  |  |  |  |  |  |  |  |  |  |  |
| --- | --- | --- | --- | --- | --- | --- | --- | --- | --- | --- | --- | --- | --- | --- | --- | --- |
|  |  |  |  |  |  |  |  |  |  |  |  |  |  |  |  |  |
|  | Age | Gender | Performance status | Stage | Metastatic sites | DCF protocol | Number of cycles | Tumor response after DCF | Metastases response after DCF | RT dose to macroscopic tumor (Gy) | RT dose to pelvis (Gy) | Tumor response after CRT | Metastasis response after CRT | Complementary treatments | PFS (months) | OS (months) |
| HPV02-29 | 60 | F | 0 | IV | liver | mDCF | 8 | PR | PR | 60 | 40 | CR | PD | CT | 9 | not reached |
| HPV02-54 | 57 | F | 1 | IV | liver, lung | mDCF | 8 | CR | CR | 59.4 | 36 | CR | CR |  | 23 | not reached |
| HPV02-61 | 63 | F | 1 | IV | bone | mDCF | 8 | PR | CR | 59.4 | 36 | PR | PD | colostomy and CT | 9 | 13 |
| HPV02-18 | 58 | F | 1 | IV | liver | sDCF | 6 | PR | CR | 59.4 | 36 | CR | CR |  | not reached | not reached |
| HPV01-31 | 75 | F | 0 | IIIB |  | mDCF | 5 | PR |  | 59.4 | 36 | CR | CR | total mesorectal excision (ypT0pN0(0 /2)R0 | not reached | not reached |
| HPV01-9 | 59 | F | 0 | IV | liver | sDCF | 6 | CR | CR | 59.4 | 50 | CR | CR |  | not reached | not reached |
| HPV01-26 | 56 | M | 0 | IV | lung | mDCF | 8 | PR | CR | 59.4 | 46 | CR | CR |  | 21 | not reached |
| HPV01-22 | 63 | F | 0 | IV | bone | mDCF | 6 | CR | PR | 59.4 | 36 | CR | CR |  | 32 | not reached |
| HPV01-11 | 57 | F | 0 | IIIB |  | sDCF | 3 | PR |  | 59.4 | 36 | CR | CR |  | 13 | 24 |
| HPV01-06 | 52 | M | 0 | IIIB |  | sDCF | 10 | PR |  | 59.4 | 36 | CR | CR |  | 20 | 50 |
| HPV02-22 | 76 | M | 1 | IV | iliac common adenopathy | mDCF | 8 | CR | CR | 59.4 | 36 | CR | CR |  | not reached | not reached |
| HPV01-29 | 69 | F | 1 | IV | liver | mDCF | 11 | PR | PR | 59.4 | 36 | PR | CR | local excision of a millimetric residual lesion | 16 | not reached |
| HPV01-32 | 51 | F | 0 | IV | liver | mDCF | 8 | PR | PR | 59.4 | 36 | CR | PD | CT | 9 | 11 |
| SCARCE 1 | 45 | F | 1 | IV | liver | mDCF | 5 | PR | PD (RC on liver but PD with new adenopathy) | 59.4 | 36 | CR | CR |  | not reached | not reached |
| SCARCE 5 | 73 | F | 0 | IIIB |  | mDCF | 8 | PR | CR | 59.4 | 36 | CR | CR |  | not reached | not reached |
| HPV01 | 47 | F | 2 | IV | skin | mDCF | 8 | PR |  | 59.4 | 36 | PR | CR | CT | 11 | 20 |

Abbreviations. F : Female ; M : Male ; DCF : Docetaxel, Cisplatin and 5-Fluorouracil ; sDCF : standard DCF ; mDCF : modified DCF ; CR : complete response ; PR : partial response ; PD : progressive disease ; CT : Chemotherapy ; RT : Radiation : CRT : Chemoradiation ; PFS : Progression-Free Survival ; OS : Overall Survival
