# Supplementary material for: Long-Term Disease Control After locoregional Pelvic Chemoradiation in Patients with Advanced Anal Squamous Cell Carcinoma
Source: Front Oncol. 2022 Jul 22;12:918271. doi: 10.3389/fonc.2022.918271 (PMC9354951; doi:10.3389/fonc.2022.918271)
Supplement: Supplementary file 2 [file Table_2.docx]

**Supplementary Table 2.** Compliance to concurrent chemotherapy

|  |  |  |  |  |  |  |  |  |  |
| --- | --- | --- | --- | --- | --- | --- | --- | --- | --- |
|  | **Mitomycin** | | **Capecitabine / 5-Fluorouracil** | | | | | | |
|  | Cycle 1 | Cycle 2 | Week 1 | Week 2 | Week 3 | Week 4 | Week 5 | Week 6 | Week 7 |
| 1 patient* | Full dose | Full dose | Full dose |  |  |  | Full dose |  |  |
| 3 patients | Full dose | Full dose | Full dose | Full dose | Full dose | Full dose | Full dose | Full dose | Full dose |
| 1 patient (neutropenia, thrombocytopenia) | Full dose | 35% DR | Full dose | Full dose | Not given | Not given | Not given | Not given | Not given |
| 1 patient | Full dose | Not given | Full dose | Full dose | Full dose | Full dose | Full dose | Full dose | Full dose |
| 1 patient (thrombocytopenia) | Full dose | Not given | Full dose | Full dose | Full dose | Full dose | Not given | Full dose | Full dose |
| 1 patient (neutropenia, thrombocytopenia, hand-foot syndrome) | Full dose | Not given | Full dose | Full dose | 30% DR | 30% DR | 50% DR | Not given | Not given |
| 1 patient (neutropenia, thrombocytopenia) | Full dose | Not given | Full dose | Full dose | 20% DR | Not given | Not given | Not given | Not given |
| 1 patient (neutropenia) | Full dose | Not given | Full dose | Full dose | Not given | Not given | Not given | Not given | Not given |
| 1 patient (pancytopenia) | Full dose | Not given | Full dose | Full dose | Not given | Not given | Not given | Not given | Not given |
| 1 patient (pancytopenia) | 25% DR | Not given | 50% RD | 50% RD | Not given | Not given | not given | Not given | Not given |
| 2 patients | Full dose | Full dose | MD | MD | MD | MD | MD | MD | MD |
| 1 patient | Full dose | Not given | MD | MD | MD | MD | MD | MD | MD |
| 1 patient | MD | MD | MD | MD | MD | MD | MD | MD | MD |
| Full dose | 14 (88) | 6 (38) | 11 (69) | 10 (67) | 5 (33) | 5 (33) | 5 (31) | 5 (33) | 5 (33) |
| Dose reduction | 1 (6) | 1 (6) | 1 (6) | 1 (6) | 2 (13) | 1 (7) | 1 (6) | 0 | 0 |
| Not administrered | 0 | 8 (50) | 0 | 0 | 4 (27) | 5 (33) | 6 (38) | 6 (40) | 6 (40) |
| Missing data | 1 (6) | 1 (6) | 4 (25) | 4 (27) | 4 (27) | 4 (27) | 4 (25) | 4 (27) | 4 (27) |

Abbreviations. MD : Missing Data ; DR : Dose Reduction

* received intra-veinous 5-Fluorouracil
