# Supplementary material for: Long-Term Disease Control After locoregional Pelvic Chemoradiation in Patients with Advanced Anal Squamous Cell Carcinoma
Source: Front Oncol. 2022 Jul 22;12:918271. doi: 10.3389/fonc.2022.918271 (PMC9354951; doi:10.3389/fonc.2022.918271)
Supplement: Supplementary file 3 [file Table_3.docx]

**Supplementary Table 3. Results from restrospective and prospective studies for chemotherapy and concurrent chemoradiation in metastatic SCCA.**

|  | N | ORR (%) | CR rate (%) | Median PFS (months) | 1-year PFS rate (%) | 3-year PFS rate (%) | Median OS (months) | 1-year OS rate (%) | 3-year OS rate (%) | 5-year OS rate (%) | Grade 3-4 toxicity (%) |
| --- | --- | --- | --- | --- | --- | --- | --- | --- | --- | --- | --- |
| Chemotherapy alone | | | | | | | | | | | |
| Faivre *et al*. 1999 (17) | 18 | 66 | 6 |  |  |  | 34 | 62 |  | 32 |  |
| Eng *et al.* 2014 (16) | 77 |  |  | 7 for all patients / 16 for multidisciplinary management |  |  | 22 for all patients / 53 for multidisciplinary management |  |  |  |  |
| InterAACT, Rao *et al.* 2018 (21) | 45 | 59 |  | 8.1 |  |  | 20 |  |  |  | 71 |
| Epitopes-HPV02, Kim *et al.* 2018 (25) | 69 | 86 | 45 | 11 | 47 |  | 36.3 | 83.1 | 51.3 |  | 70 |
| Pooled Epitopes-HPV01 and Epitopes-HPV02, Kim *et al*. 2020 (19) | 115 | 87.7 | 40.3 | 12.2 | 50.7 | 26 | 50.2 for mDCF 36.3 for sDCF |  | 56.5 (mDCF)/ 51.5(sDCF) | 44 | 53 for mDCF 83 for sDCF |
| Chemotherapy and local treatment | | | | | | | | | | | |
| Evesque *et al.* 2017 (40) | 50 | 74 | 10 | 6 |  |  | 20 |  |  |  | 37 |
| Holliday *et al*. 2018 (41) | 30 |  |  | 0.9 |  | 42 |  |  | 67 |  |  |
| Sclafani *et al.* 20019 (42) | 8 |  |  | 5 |  |  | 31 |  |  | 25 |  |
| Abdelazim *et al.* 2019 (33) | 978 |  |  |  |  |  | 20 for CRT-group / 16 for CT-alone |  |  | 22 for CRT-group / 15 for CT-alone |  |
| Wang *et al.* 2019 (43) | 1475 |  |  |  |  |  | 21.2 for CRT-group / 15.6 for CT-alone |  |  | 26 for CRT-group / 12 for CT-alone |  |
| Our study | 16 | 100 | 69 | 14.9 | 62.5 | 33 | Not reached | 87.5 | 75 |  | 73 |

Abbrevations : N : number of patients ; ORR : Objective Response Rate ; CR : complete response ; CRT : Chemoradiotherapy group ; CT : Chemotherapy PFS : Progression-Free Survival ; OS : Overall survival ; DCF : Docetaxel Cisplatine 5 Fluorouracil ; mDCF : modified DCF ; sDCF : standard DCF
